# Addressing transportation barriers in oncology: existing programs and new solutions

**DOI:** 10.1007/s00520-024-08514-2

**Published:** 2024-04-29

**Authors:** Sophia Pringle, Emily M. Ko, Meredith Doherty, Anna Jo Bodurtha Smith

**Affiliations:** 1 Leonard Davis Institute of Health Economics, Saint Joseph’s University, Philadelphia, PA USA; 2 Division of Gynecologic Oncology, Department of Obstetrics and Gynecology, University of Pennsylvania Health Systems, Philadelphia, PA USA; 3 Leonard Davis Institute of Health Economics, University of Pennsylvania Health Systems, Philadelphia, PA USA; 4 Penn Center for Cancer Care Innovation, Abramson Cancer Center, University of Pennsylvania Health Systems, Philadelphia, PA USA; 5 School of Social Policy and Practice, University of Pennsylvania, Philadelphia, PA USA

**Keywords:** Transportation, Health disparities, Clinical trials, Cancer

## Abstract

Transportation is an underrecognized, but modifiable barrier to accessing cancer care, especially for clinical trials. Clinicians, insurers, and health systems can screen patients for transportation needs and link them to transportation. Direct transportation services (i.e., ride-sharing, insurance-provided transportation) have high rates of patient satisfaction and visit completion. Patient financial reimbursements provide necessary funds to counteract the effects of transportation barriers, which can lead to higher trial enrollment, especially for low socioeconomic status and racially and ethnically diverse patients. Expanding transportation interventions to more cancer patients, and addressing knowledge, service, and system gaps, can help more patients access needed cancer care.

Transportation is an underrecognized, but modifiable barrier for up to one-third of cancer patients, leading to delayed or missed appointments and poorer health outcomes [1]. Transportation barriers include distance to treatment, physical mobility, reliable vehicle access, available public transportation, having a caregiver to drive, and affordability of transportation (e.g., gas money, bus fare). Furthermore, side effects of cancer treatment, such as blurred vision or fatigue, may limit one’s ability to drive, leading to new development of transportation barriers. Transportation barriers have a significant influence on patients’ decision to terminate or continue cancer treatment and their likelihood of receiving optimal care [2]. In cervical cancer, for example, US patients were proportionally less likely to receive guideline-concordant treatment as distance traveled for care increased [3]. In this commentary, we describe the impact of transportation barriers on cancer care, using clinical trials as an example, and evidence-based interventions to support patients with transportation needs with a US focus. We performed a PubMed search using terms for clinical trials, cancer, and transportation in July 2022 for this scoping review.

## Clinical trials and transportation

Transportation barriers directly limit clinical trial enrollment. Clinical trials typically require more visits, adding time and transportation costs [4, 5]. In a study of US lung cancer patients, 7% reported transportation barriers as the reason for not enrolling in an eligible clinical trial [6]. Another study found that 27% of trial-eligible, non-enrolled patients reported transportation barriers [5]. Moreover, transportation barriers disproportionately affect historically marginalized populations, such as low-income or Black patients, who are also under-enrolled in clinical trials [7, 8]. Only 3–5% of cancer patients participate in clinical trials with the majority of trial enrollees being white and higher income [9, 10].

## Interventions to reduce transportation barriers to cancer care

Interventions at the clinician, insurer, and system level can help address transportation barriers. Medicaid, for example, provides non-emergency medical transportation (NEMT) to and from appointments, serving more than 4 million patients (10% of enrollees) annually [11]. NEMT ranges from reimbursement for public transportation, direct transportation via medical vans or ambulances, or patient reimbursement for miles traveled in their own car. NEMT is currently limited to 25 miles for most patients (75 miles for rural), limiting utility for patients who live farther from care [12]. Like other components of Medicaid, NEMT varies between states. Five states require co-pays of $1–2 per every one-way ride [13]. For a patient undergoing curative intent weekly chemotherapy and daily radiation for locally advanced cervical cancer, $1 per one-way ride easily translates into $100 or more over 2 months, a financial obstacle for low-income patients. Other states require physician prescription for services or limit the total number of rides. Some Medicare Advantage plans cover transportation, but traditional Medicare fee-for-service covers only cover emergency ambulance transportation [14]. Few private or employer-sponsored insurers—the type of insurance covering the majority of Americans—provide transportation benefits.

Another potential option, not currently covered by public or private insurance, is transportation network companies (more commonly known as rideshare companies). Rideshare services that are accessible through companies like Uber and Lyft, can be compliant with Health Insurance Portability and Accountability Act, and allow clinical teams to schedule rides. One study using rideshare in gynecology found high patient and clinician satisfaction, with 94% of visits being attended, and patients spending half the travel time with ridesharing that they would have on public transportation [15]. Without insurance coverage, rideshare services vary nationwide as funding is dependent on philanthropy and institutional contracts. Where ridesharing is available, patient navigators can connect individuals to transportation services [5].

In addition to system-level transportation programs, patient-level financial reimbursement can reduce transportation barriers. Financial reimbursement may include bus fare, plane tickets, gas, taxi vouchers, and hospital parking that can be prepaid or paid retrospectively. Such programs are particularly valuable for clinical trials where patients are more likely to be traveling farther for care with longer and more frequent appointments. In a trial of financial reimbursement for cancer clinical trials at the University of California-San Francisco, a majority (75%) of patients traveled more than 3 hours with about 17% traveling more than 8 hours for treatment [16]. A similar program was piloted at the University of Pennsylvania Abramson Cancer Center, using retrospective financial reimbursement for travel-related expenses [17, 18]. Both programs found that financial reimbursement for travel costs was associated with higher trial enrollment and greater enrollment of low socioeconomic status and racially and ethnically diverse patients [16, 18].

## Limitations of current transportation interventions

Transportation interventions reduce barriers to cancer care, but limitations need to be addressed for services to become widespread and accessible. The first limitation is a knowledge gap: many patients do not know they qualify for transportation through Medicaid or select Medicare plans [19]. Cancer centers, supportive care programs, and clinicians can educate patients about transportation resources. The second limitation is a service gap. Current transportation services are frequently inaccessible to individuals who use wheelchairs, are bed-bound, or require ramps, meaning there is not enough paratransit coverage in the US and especially in low- and middle-income countries. The third limitation lies in a system gap. Transportation interventions are only effective if services are financially supported and sustainable. Without insurance coverage, transportation services are limited geographically and dependent on donor and grant funding for virtually all non-Medicaid patients. Volunteer driver programs, such as American Cancer Society’s Road to Recovery, or shuttles, serve only a fraction of patients in need [20]. Clinical trials could budget for transportation costs to offset greater transportation burden of clinical trial participation.

Implementation of rideshare programs and financial reimbursement programs can improve cancer care. Greater diversity in clinical trials is necessary for generalizability and health equity. Addressing transportation barriers starts with screening. Clinicians can break down barriers by asking patients if they need transportation and connecting patients with supportive care resources. Clinics should screen for transportation and other social determinants of health. For Medicaid NEMT, removal of mileage limits and state uniformity would improve transportation. Medicare and private insurers could follow Medicaid, providing transportation services to and from outpatient appointments. Transportation helps patients obtain timely cancer care, which could be cost-effective for insurers [21, 22]. We should scale and study transportation interventions to mitigate the impact of transportation barriers on cancer care.

## References

[1] Wercholuk AN, Parikh AA, Snyder RA (2022). The road less traveled: transportation barriers to cancer care delivery in the rural patient population. JCO Oncol Pract.

[2] Etminani-Ghasrodashti R, Kan C, Mozaffarian L (2021) Investigating the role of transportation barriers in cancer patients’ decision making regarding the treatment process. 2675(6):175–187. 10.1177/0361198121991497

[3] Nasioudis D, Musselman K, Gordhandas S (2020). Disparities in the use of adjuvant external beam radiation therapy in node-positive cervical cancer patients following hysterectomy. Am J Clin Oncol.

[4] Coakley M, Fadiran EO, Parrish LJ, Griffith RA, Weiss E, Carter C (2012). Dialogues on diversifying clinical trials: successful strategies for engaging women and minorities in clinical trials. J Womens Health (Larchmt).

[5] Cartmell KB, Bonilha HS, Simpson KN, Ford ME, Bryant DC, Alberg AJ (2020). Patient barriers to cancer clinical trial participation and navigator activities to assist. Adv Cancer Res.

[6] Baggstrom MQ, Waqar SN, Sezhiyan AK (2011). Barriers to enrollment in non-small cell lung cancer therapeutic clinical trials. J Thorac Oncol.

[7] Smith AJB, Alvarez R, Heintz J, Simpkins F, Ko EM (2023). Disparities in clinical trial participation in ovarian cancer: a real-world analysis. Gynecol Oncol.

[8] Smith AJB, Powell K, Doherty M (2023). Transportation assistance in gynecologic oncology: a pilot study. JCO Oncol Pract..

[9] Murthy VH, Krumholz HM, Gross CP (2004). Participation in cancer clinical trials: race-, sex-, and age-based disparities. JAMA.

[10] Aldrighetti CM, Niemierko A, Van Allen E, Willers H, Kamran SC. Racial and ethnic disparities among participants in precision oncology clinical studies. JAMA Netw open. 2021;4(11). 10.1001/JAMANETWORKOPEN.2021.33205 10.1001/jamanetworkopen.2021.33205 PMC8576580 34748007

[11] Medicaid and CHIP Payment and Access Commission (2021) Report to congress on medicaid and CHIP. pp 153–197. https://www.macpac.gov/publication/june-2021-report-to-congress-on-medicaid-and-chip/#:~:text=MACPAC's%20June%202021%20Report%20to,and%20behavioral%20health%20care%20through

[12] Pancoe S, Ko EM, Smith AJB (2024). Federal transportation regulations limit access to clinical trials and oncology care. JAMA Oncol Published online.

[13] Kaiser Family Foundation. Medicaid benefits: non-emergency medical transportation services. Accessed September 7, 2023. https://www.kff.org/medicaid/state-indicator/non-emergency-medical-transportation-services/?currentTimeframe=0&sortModel=%7B%22colId%22:%22Location%22,%22sort%22:%22asc%22%7D

[14] Ambulance services coverage. Accessed September 7, 2023. https://www.medicare.gov/coverage/ambulance-services

[15] Vais S, Siu J, Maru S (2020). Rides for refugees: a transportation assistance pilot for women’s health. J Immigr Minor Heal.

[16] Borno HT, Zhang L, Zhang S, et al. Implementation of a multisite financial reimbursement program in cancer clinical trials integrated with patient navigation: a pilot randomized clinical trial. JCO Oncol Pract. Published online February 23, 2022:OP2100328. 10.1200/OP.21.00328 10.1200/OP.21.00328 PMC9191303 35196064

[17] Gerber DE, Tiro JA, McNeill LH, et al. Enhancing access to and diversity in cancer clinical trials through a financial reimbursement program: protocol to evaluate a novel program. Contemp Clin Trials. 2022;121. 10.1016/J.CCT.2022.106922 10.1016/j.cct.2022.106922 36096281

[18] Penn’s Abramson Cancer Center doubles the percentage of Black participants in clinical trials - Penn Medicine. Accessed September 7, 2023. https://www.pennmedicine.org/news/news-releases/2021/may/penns-abramson-cancer-center-doubles-the-percentage-of-black-participants-in-clinical-trials

[19] Becerra X (2023) Expanded report to congress non-emergency medical transportation in Medicaid, 2018–2021. https://www.medicaid.gov/medicaid/benefits/downloads/nemt-rtc-2018-2021.pdf

[20] Be a road to recovery volunteer | American Cancer Society. Accessed February 28, 2024. https://www.cancer.org/involved/volunteer/road-to-recovery.html?utm_source=google&utm_medium=cpc&utm_campaign=road_to_recovery_search&utm_source=bing&utm_medium=cpc&utm_campaign=Unassigned&utm_term=volunteertodrivecancerpatients&gad_source=1&gclid=EAIaIQobChMI0qXQ6frOhAMVAGUPAh32qQX7EAAYASAAEgLhKvD_BwE

[21] Nipp RD, Lee H, Powell E (2016). Financial burden of cancer clinical trial participation and the impact of a cancer care equity program. Oncologist.

[22] Vais S, Thomson L, Williams A, Sobota A (2020). Rethinking rideshares: a transportation assistance pilot for pediatric patients with sickle cell disease. J Health Care Poor Underserved.

